# Views of family physicians on heterosexual sexual function in older adults

**DOI:** 10.1186/s12875-018-0770-1

**Published:** 2018-06-11

**Authors:** Inbar Levkovich, Ateret Gewirtz-Meydan, Khaled Karkabi, Liat Ayalon

**Affiliations:** 1 The Division of Family Medicine, Department of Family Medicine, The Ruth & Bruce Rappaport Faculty of Medicine, Technion-Israel Institute of Technology, 6 Hashachaf St., Bat-Galim, 35013 Haifa, Israel; 2 The Louis and Gaby Weisfeld School of Social Work, Bar-Ilan University, Ramat Gan, Israel; 3 Department of Family Medicine, The Ruth & Bruce Rappaport Faculty of Medicine, Technion-Israel Institute of Technology, Clalit Health Services, Western Galilee District, Haifa, Israel

**Keywords:** Sexuality, Primary care, Psychogeriatrics, Health aging, Aging

## Abstract

**Background:**

Sexual functioning among older adults has received little attention in research and clinical practice, although it is an integral part of old age. As older adults tend to consume health services and to visit family physicians more frequently, these care-providers serve as gatekeepers in the case of sexual concerns. The present study evaluated the perceptions of family physicians regarding sexuality in older adults.

**Method:**

Qualitative interviews with 16 family physicians were conducted. We used in-depth, semi-structured interviews.

**Results:**

Three main themes emerged: 1. Family physicians described having difficulty in raising questions about sexuality to older patients. 2. Family physicians tended towards the biological side of the spectrum, focusing on the patient’s medical problem and asking physiological questions. 3. Family physicians mainly related to medication administered to their male patients, whereas a minority also described the guidance they provided to older individuals and couples.

**Conclusions:**

The study shows that family physicians tend not to initiate discourse with older patients on sexuality, but rather discuss sexuality mostly in conjunction with other medical conditions. Implications for research and practice are discussed.

## Background

Sexuality is an important part of human health and well-being over the life cycle and in old age [1–3]. Sexuality represents people’s sexual interest in and attraction to others as well as their capacity to have erotic experiences [4]. An individual’s sexuality includes his or her attitudes, values, knowledge and behaviors [5]. Sexuality is different from sex. Sexuality is a much broader term, has many components, and includes much more than sexual intercourse [4, 6]. Sex, on the other hand, is a biological construct that encapsulates the anatomical, physiological, genetic, and hormonal variations that exist in species [6]. “Older adults” are traditionally defined as being over the age of 65 [7, 8]. The classified respondents between the aged 65–74 as “young old”, aged 75–84 as “old old”, and those aged 85 and older as “oldest old” [7, 8].

Sexual function and sexual satisfaction among the older adult population have received little attention in research and clinical practice, although they are an integral part of old age and despite the fact that this age group may be characterized by sexual changes [9, 10]. Studies have shown that many older adults are interested in engaging in sex and are sexually active in their later years [11]. Surveys conducted in several countries have found that older adults attest to the importance of sexual activity in their lives and the sense of security it provides [12–14]. A study in which 3000 older adults were interviewed found that although the level of interest in sexuality was lower among older adults, 59% of participants aged 75–85 reported the importance of sexuality to their lives [11].

Studies indicate that the prevalence of symptoms and complaints increases with age - as much as double in comparison to young people [15]. It was also found that as age advances, there is a diminished interest in sexuality, and sexual activity is less frequent in comparison with younger ages [11, 16]. In interviews with 44 people aged 50–92, it was found that adults over 70 were less interested in sexuality in comparison with younger adults in this age range [16]. In a cohort study spanning 10 years, data from 3032 respondents aged 25–74 were analyzed; the results showed that, among men, sexual interest remained stable across age groups whereas women’s interest declined. Thus, the literature suggests that desire does not always decline as men and women age [17]. It is, rather, a complex component which is influenced by many variables [13].

One such variable concerns physiological and biological changes that occur with age.

It has been suggested that it is not age that causes the decline in sexual activity, but rather the natural physiological changes and common health problems that accompany older adults [16]. Hormonal changes associated with older adults are mainly reflected in the slowing down of sexual response and a decrease in the intensity of sexual arousal and pleasure. Among women, a decline in estrogen levels, which is characteristic of the post-menopausal period, causes atrophy (e.g, dryness, burning, dyspareunia) of the external female organs and a decrease in orgasm intensity [9]. As a result, women experience post-menopausal emotional changes [18]. The most prevalent sexual problems among women were low desire (43%), difficulties related to vaginal lubrication (39%), and inability to climax (34%) [17]. Women expressed less interest in non-sexual activities such as holding hands, kissing, hugging, and in masturbating or sexual activity [19]. After the age of 50, more women (56.6%) reported a cessation in sexual activity at some time, due to various reasons compared to men (16.6%), who remained sexually active (83.4%) [19].

In men, free testosterone decline causes a slowdown in response to sexual arousal, which consequently leads to a need for stronger, longer-lasting physical stimulation in order to achieve an erection. Penile erection is not as firm as in younger age groups, the amount of sperm lessens and there is a decrease in the intensity of ejaculation. In addition, there may be a decrease in sex drive [20] and endocrinal changes (‘male menopause’) [21]. There are many biomedical reasons for erectile dysfunction (ED), such as medication [22], diseases and surgeries [23], diabetes [24], and cardiovascular problems [25]. However, contrary to general opinion, Mazur and others [26] were unable to find a direct link between sexual desire and low free testosterone levels or depression in older men.

Although these changes are valid and likely affect the sexual function of older men and women, other factors are also important. The biopsychosocial model [17, 18] supports the approach that biological, psychological (including thoughts, emotions, and behaviors) and social factors affect one’s health condition. The model promotes the perception of the human being as a whole, whereby the body and mind are interconnected and are in constant interaction with the social environment [27, 28]. Hence, in addition to medical conditions and health problems, emotional factors, such as depression and anxiety, affect sexual activity at any age, but increase in frequency as aging progresses [22]. Meaningful life events such as retirement, death of a loved one, absence of a spouse and loss of privacy following relocation to an institutionalized setting are some of the causes of sexual disorders among older adults [2, 21].

Healthcare providers, including physicians, nurses, social workers, and psychologists, are required to understand the complexity of the psychological and biological factors that affect sexual function in old age, in order to help older adults cope with issues concerning sexuality [21]. Because older adults tend to consume health services and to visit family physicians more frequently [29, 30], these care-providers serve as gatekeepers in the case of sexual concerns [29, 30]. Little research has been conducted about older people’s experiences of talking to family physicians about sexual function. Most participants said they did not discuss sexual issues with their healthcare providers, and the physician did not raise the issue [3]. For those individuals who did discuss sexual with their healthcare providers, negative and stigmatizing responses were common [31]. Barriers to discussing sexual issues with one’s physician include personal embarrassment, lack of knowledge and awareness, and fear of wasting the doctor’s time [16, 32]. Others said they did not consider sexual dysfunction to be a medical problem or a problem that could be treated by a doctor [32].

Other barriers to discussing sexual issues with older adults concern the physicians. Studies reporting family physicians’ knowledge of sexuality in old age were inconsistent. Whereas some studies indicate appropriate or adequate knowledge, others show limited knowledge among family physicians in this field [33–35]. Moreover, the average medical staff’s personal sense of comfort and confidence to discuss old-age sexuality with patients is low [36]. Additional barriers to discussing sexual concerns are lack of time, lack of communication skills and a general desire to avoid the subject [33, 37]. Health care professionals often assume that sexual function in older adults is beyond the scope of their expertise [4]. For instance, family physicians discuss sexual issues more often with young people than with older adults, and may regard sexuality as an intimate, private subject that should not be discussed in old age [38–40], thereby expressing ageist attitudes towards older adults.

An understanding of the impact of sexual orientation on older adults’ life experiences may assist healthcare professionals in their efforts to determine appropriate interventions. Until recently, the literature has tended to disregard the sexual orientation of older adults. For example, many studies were framed from a heteronormative perspective, while very few studies have explored older lesbian, gay, bisexual, transgender, queer and intersex individuals (LGBTQ&I) [41]. Many LGBTQ&I older adults have built vibrant communities and a sensibility that they can count on each other [41]. Many LGBTQ&I older adults have also created close, intimate families of choice, comprised of loved ones, including current and former partners and friends [42]. In the Gay and Gray Project [43], over three-quarters of the respondents reported having active sexual lives and over half felt their sexuality had an important positive impact on their lives. Yet, population estimates suggest that one-third to one-half of older gay and bisexual men live alone, without adequate services or support [44, 45]. In the CAP project, 61% of the gay and 53% of the bisexual male participants reported experiencing loneliness [44]. Older LGBTQ&I adults face unique issues that can impede their well-being [44]. In a mixed methods study, participants identified that both ageism and heterosexism presented challenges when attempting to secure adequate housing and receive emotional support. Legal issues were another identified source of primary concern for the elderly with regard to a lack of legal protection for “married” same sex couples compared to opposite sex couples [46]. Family physicians may ignore LGBTQ&I needs and preferences due to discomfort, uncertainty or lack of LGBTQ&I -specific health knowledge [47–51]. This might reinforce heteronormative status quo and stereotypes [52].

Because family physicians are the main *gatekeepers* for a variety of medical and psychosocial issues concerning older adults [53] and in light of the lack of empirical knowledge concerning the way physicians perceive sexuality in older adults, the goal of this qualitative study was to investigate the perceptions of family physicians regarding sexuality in older adults.

## Method

The study used a qualitative-phenomenological approach [54]. This approach attempts to obtain an in-depth understanding of the phenomenon by entering the world and experiences of the participants. The qualitative-phenomenological research approach was chosen to enable family physicians to tell their stories and give meaning to their experiences. The descriptive power of this approach allows for an in-depth understanding of the family physicians’ perceptions of sexuality in older adults. Such research is based on small samples composed of a limited number of ‘information-rich’ informants, where depth is exchanged for representativeness [55, 56].

### Sample and population

This study was supported by a grant from the Israel National Institute for Health Policy Research No. 16/2016/ℵ. The present study focused on a sample of family physicians. Recruitment occurred via emails sent to family physicians displayed in a variety of health clinics. Physicians were offered to participate in the study after receiving a comprehensive explanation of the study’s purpose. Participants were 16 family physicians, aged 36–64. The majority were born in Israel; 13 worked in ‘Clalit Health Services’ and three worked in ‘Maccabi Healthcare Services’, the largest and second-largest health funds in Israel, respectively. Half of the participants were women. Seven physicians worked in urban clinics; the other nine worked in rural clinics.

### Instrument

The data were collected using in-depth, semi-structured interviews. We designed the interview guide with questions to allow the participants to share their stories openly. The interview guide included several questions: “Tell me about sexual function in older adults;” “What are your reactions (thoughts, feelings, behaviors) when patients consult you about sexual function in older adults?”; “How do you handle sexual dysfunction in older adults?”; “What are the advantages and disadvantages of the treatment given to alleviate sexual dysfunction?”; “How do sexual difficulties among older adults differ from those of younger adults?” The interviewer encouraged physicians to narrate their experiences in their own words [57] Table 1.

**Table 1:** Interview questions posed to family physicians for the qualitative analysis

1	How do you define sexuality?
2	Tell me about sexual function in older adults.
3	Do the reasons for engaging in intimate relations differ between young adults and older adults?
4	In your opinion, what reasons might elderly individuals have for refraining from engaging in intimate relations?
5	In your opinion, which factors might influence the levels of sexual function and sexual satisfaction in older adults?
6	How do you think society/the media perceives sexuality among the elderly?
7	How does treatment of sexual function differ between younger adults and older adults?
8	Tell me about contacting/referring patients to different specialists in relation to sexual function difficulties among the elderly.
9	What are the advantages/disadvantages of contacting different specialists in relation to sexual dysfunction in older adults?
10	In your opinion, how is it possible to create open lines of communication about sexual function between elderly patients and their physicians?

### Procedure

The Meir Medical Center Hospital Ethics Committee approved study No. 0262–16-MMC. The researchers identified the participants and requested their written consent to participate in the study. Prior to conducting the interviews, interviewers were required to undergo a reflection process [58], including the ability to reflect on the identities, social locations, assumptions, and life experiences they bring to the research endeavor, along with their interactions with interviewees. For example, the interviewers were asked how they identify themselves in relation to the subject - sexuality in older adults – and were requested to express their emotions, attitudes and opinions. Strong relational skills and competence in self-reflexivity helped to ensure that interviewers were authentic, attentive, able to critically examine their own reactions and responses, and address any awkward moments that might arise in the interview interaction.

The researchers identified the participants and requested their written consent to participate in the study. The participants then underwent an in-depth interview in their clinics or homes. Interviews were conducted in Hebrew, and lasted approximately 1 hour. Each interview was tape-recorded and later transcribed verbatim. At the end of the all the interviews, we performed the data analysis.

### Data analysis

We used content analysis in this study. Where appropriate, we paraphrased and generalized respondents’ statements. We organized similar passages by topic in order to identify individual influential factors, and then combined similar factors into major categories, to identify the main themes. Content analysis is used to unobtrusively explore large amounts of textual information, so as to determine trends and patterns of words used their frequency, and their relationships. Data analysis consisted of the following stages: 1. We first read all the data several times, to achieve immersion and obtain a sense of the text as a whole. 2. Open coding: The researcher (I.L.) first read each interview transcript line-by-line, jotting down notes to capture and identify initial units of meaning emerging from the data, and to allow the subthemes and their names to flow from the data [55]. 4. The researcher (L.A.) then reviewed the larger themes and discussed them with I.L. 4. Axial coding: The researchers gradually detected context and content-related associations between themes and sub-themes. Then, they compared all of the interviews to consolidate meaning and named the themes. Next, the researchers examined the interrelationships among the initial codes and sorted them into higher-order theoretical codes [55]. 5. Integration: The core themes that emerged from the data were reordered conceptually and placed back into context, enabling the analysis and integration of large amounts of data and the generation of abstractions and interpretations [59].

Reflecting on our experiences, we established biases, and prejudices regarding sexuality, older adults and family physicians [60, 61] during all the study’s stages. The researcher (I.L.) interviewed the family physicians, sharing her thoughts and feelings which arose during the interviews and data analysis. For example: initiated by some family physicians’ attitudes about sexuality in old age, she expressed a feeling of unfairness concerning the way women are treated by family physicians with regard to sexuality.

## Results

Analysis of the qualitative interviews identified three main themes. The themes can be placed on a continuum of psychosocial factors at one end vs. biological factors at the opposite end, with most physicians leaning towards the biological end of the continuum as they initiate conversations with patients, perform diagnoses or recommend treatment for sexual dysfunction in older adults.

The first theme addressed the difficulty of asking questions related to sexuality: **“I hear nothing because I ask nothing”; family physicians refrain from initiating a conversation about sexuality with older adults** - Family physicians described having difficulty in raising questions about sexuality with their older patients, due to work overload and the lack of time; they also felt that this issue was not their top priority when talking with these patients and that questions concerning such intimate issues might harm their relationship with the patient. In those cases where they felt at ease asking questions about sexuality, it was done when sexuality was seen as being part of a variety of medical conditions, so that the biological-medical model guided them. **The second theme dealt with the challenge involved in the diagnosis: “Trying to understand where the difficulty lies”** - Family physicians described that most sexuality-related visits were made by males and the main complaint was erectile dysfunction. A variety of diagnostic questions focusing on the physiological aspects were described, including a physical examination. Only a small number of women turned to their family physicians; some came to receive medication for their husband or described gynaecological problems, from which a discourse on sexual function ensued. The third theme that emerged was: **“There are medications that can be offered”;** treatment of sexual function in older adults - Family physicians mainly described medication administered to men, and a minority also described counselling, which included individual or couple’s guidance to coordinate mutual expectations, encourage intimacy and referral for further treatment. However, the predominant view was that the main treatment which can be offered to patients is pharmacological.

**Theme 1: “I hear nothing because I ask nothing”; family physicians refrain from initiating a conversation about sexuality with older adults.**

Most of the family physicians who were interviewed said they do not ask older patients about sexual functioning and sexual difficulties on a regular basis. Many family physicians explained that the focus of the doctor-patient encounter is medical and other topics receive a lower priority. Much of the physician’s attention is focused on illnesses, medication and diagnosis; therefore, the subject of sexual problems is often perceived as being less significant. Some family physicians emphasized the importance of the subject, but noted that in regard to older adults, a range of higher-priority medical issues require attention and should be examined. These physicians expressed a preference for the biological approach within the model, which attributes a rather limited importance to the psychosocial parts:

“I *feel that it (sexual dysfunction) is very important, but it always gets lost among all the other issues and diseases and things that need to be done... the mammography and the other tests, and the drugs… it is very often neglected”. (Family physician 5)*

Family physicians have reported certain limitations that prevent them from asking about sexual function: workload, time constraints, and fear of offending patients were mentioned as the main factors. The physicians described the health system as demanding and the available resources as significantly inadequate. Anger, frustration, and even despair were expressed regarding the limited time available for the physician-patient interaction. In addition, physicians wondered whether questions about sexuality would not be perceived as an insensitive violation of the physician-patient relationship, with little time dedicated to each session, and the physician’s sense of inability to create the necessary intimacy and closeness needed to facilitate a discourse on these personal issues:

*“If someone makes an appointment, waits for 2 weeks and has a list of four or five medical problems, while other people are waiting outside, and we barely have 10 minutes, then how can I casually ask: How is your sexual function?” (Family physician 2)*

Most family physicians explained that discourse with older patients on sexual function and satisfaction usually takes place with those patients who suffer from chronic disorders. The catalyst for the conversation is the patient’s state of health, the common illnesses for which the patient came in or the medication he or she takes or is expected to take. Because of the barriers to discussing the subject described by the physicians, a sense of relief and confidence was described when the conversation revolved around the physician’s areas of strength, i.e., illnesses and medications, and those which he or she felt were within his or her field of expertise. These physicians tended towards the biological side of the model:

“*Yesterday, I saw an older patient, who came in only because he wanted to stop taking his diabetes medication because his diabetes had stabilized, and he had been diabetic for a very long time. Incidentally, he also mentioned a prostate problem...he wanted to undergo a protein test, and this opened up a conversation on sexual function”. (Family physician 6)*

A minority of family physicians described the catalyst for sexual questions in older age not as the medical condition, but as the patient’s emotional state. These physicians said that once they identify emotional distress or somatisation among patients, they ask them about sexual function. In their view, sexual dysfunction is not purely organic; it has emotional implications or may be caused by the patient’s emotional distress. Emotional distress may contribute to sexual-function difficulties; therefore, questions and clarifications should be initiated on this issue as well. While these physicians emphasized the model’s psychosocial aspects in the context of sexuality, they also described barriers which prevented them from asking directly and openly about sexual function, because they felt that such a conversation was less appropriate within the physician-patient encounter:

*“A 70-year-old woman with a lot of somatization…and I could understand that much of it was related to the tension at home. But when I delicately tried to ask her whether anything might be bothering or troubling her daily, it [sexual functioning] didn’t come up. I imagine it’s a very difficult thing to talk about…so I didn’t ask her directly about her sex life… I felt it would be too intrusive, invasive and painful”. (Family physician 1)*

**Theme 2: “Trying to understand where the difficulty lies”; diagnosis of sexuality in** older adults Diagnosis, which constitutes an essential component in patients’ assessment, helps physicians focus on relevant clarification of the illness, e.g., the reason for referral to treatment, current symptoms, past illnesses, etc. Within the present research, family physicians described collecting diverse patients’ diagnoses, along a biological-psychosocial continuum.

Most family physicians tended towards the biological end, preferring to focus on medical problems, and ask physiological questions, sometimes including a physical examination. The physicians described the manner in which they ask the patient which symptoms he or she is experiencing and for how long: Is it a libido problem or erectile dysfunction? Does he have a morning erection? Which medication(s) is he/she taking? What are his or her illnesses and risk factors? It seems physicians felt more at ease diagnosing on the basis of a variety of questions, similar to the way they diagnose any other illness, thoroughly and in a way which avoids dealing emotionally with sexual dysfunction:

“*So, I ask them what we’re dealing with, how long…or what problems they have… If there are also urination problems, since that’s more prostate-related, a clarification can be made in this direction…However, many times it’s just the erection [problem]…either they don’t have a morning erection, or the erection is too short”. (Family physician 4)*

Although most interviewees were more inclined towards the biological aspect, some showed a tendency towards the psychosocial aspect, examining the patient and his or her world as a whole, including family, marital and personal issues, which the patient brings from his or her world and may affect his or her condition.

A minority of the physicians focused on the patient’s intimacy issues and checked whether the patient was in a relationship, having sex, whether any changes had occurred in the relationship, and which spouse complained about a difficulty. Their questions made room for exploring the medical and physiological aspects, although this was considered a secondary priority and came after a variety of generic questions. These physicians perceived the comprehensive clarification as being deep and thorough; some described a number of sessions in which they conducted a preliminary diagnosis and later invited the couple in order to ascertain a more complete idea of the situation, thereby eventually reaching the root of the problem. These physicians described the diagnosis as starting with questions on intimacy, rather than the patient’s physiological state, and only afterwards asking about the patient’s health condition, as needed:

“*I always start out by asking about the couple’s intimacy, how they are together. Do they still sleep in the same bed? Are they still having sex? I do this gradually, in order to get an idea about their current status”. (Family physician 6)*

**Theme 3: “There are medications that can be offered”,** treatment of sexual function in older adults.

Most family physicians reported perceiving treatment of sexual function in older adults as a pharmacological treatment administered to men suffering from impotence. The physicians described a typical encounter in which the patient asks for medication on his or her own initiative or describes a related difficulty, followed by a short diagnosis, after which treatment is offered. The physicians discuss the treatment’s advantages and disadvantages with the patient and make adjustments in case the patient is taking additional medication. With the exception of patients with complex problems, whose treatments include implants and injections, the physicians described the treatment as a one-time session, followed by the automatic prescription of medication:

*“Viagra and Cialis. …Viagra isn’t really a regular treatment. You can take it whenever you feel like it…but you have to know that you are about to have sex, so you need to take it ahead of time. You have to prepare. And even if a man doesn’t have the desire, he’ll still get an erection; and if he doesn’t have sex it [the erection] will be maintained…Some other medications should be avoided, especially by coronary heart disease patients. You have to be a bit careful...”. (Family physician 4)*

Some family physicians stated that women seek treatment for sexual dysfunction in old age significantly less frequently than men. The treatment of women also focused on the biological aspect, including the prescription of medication and locally-applied ointments:

“*For women, local vaginal estrogen therapy is very effective. In cases of vaginal dryness and other complaints, I would suggest this treatment around menopause and up to a very old age – even to patients who haven’t received it previously (*why is this mentioned*?). I also prescribe this treatment for patients aged 70 and up. This usually helps significantly. In many cases, once the physical complaints and symptoms improve, desire also increases”. (Family physician 5)*

A minority of the interviewed family physicians described care of patients with sexual dysfunction as a legitimate part of their role, advising couples to prolong foreplay and be more receptive to each other’s needs, in an attempt to reduce the tension associated with penetration and reinforce intimacy. Some of the physicians described the difficulty of conducting these types of counseling sessions, claiming they are not qualified psychologists, and mentioning the short meeting time available in light of the patients’ great needs. The physicians also said they usually postpone visits with patients with whom they would like to discuss the issue of sexual function to the end of the day, or ask them to schedule a double appointment, which will allow time for a more in-depth discussion:

*“I talk about prolonging the foreplay, being more receptive to each other’s needs… reducing the stress surrounding sexual function…placing more emphasis on intimacy, the needs of the other partner…and reducing the focus on penetration. Sex is not only penetration, there’s a lot more between foreplay and penetration”. (Family physician 12)*

Referral to sexual therapy occurred in rare cases; physicians argued that most patients either are not interested or the physicians themselves do not suggest it. In more complex patients, such as those with implants and injections, physicians prescribed longer sessions, consultation with other specialists and referral to sexual therapy. Many physicians described the limitations of sexual therapy, in terms of the mobility of older adults: the distance between home and clinic and the high costs. Hence, most of the treatment options remain in the realm of the family physician.

## Discussion

The study’s objective was to expand the understanding of family physicians’ perceptions regarding sexuality among older adults.

The current study shows that most family physicians do not initiate discourse on this subject and discuss sexuality mostly in relation to common illnesses. Most physicians tended to perform a diagnosis that focused on the physiological aspects, discussing the symptoms, prevalence of the disorder, medication, other illnesses, etc. A minority of physicians examined intimacy and marital relations as an integral part of the diagnosis. The proposed treatment entailed mostly drug therapy for men, while a small number of family physicians said they discussed the couple’s sexual expectations and their difficulties, and attempted to reduce the stress associated with penetration.

The family physicians who participated in this study tended not to initiate a discussion on sexual matters with their older patients. This finding supports previous research that indicated medical staffs’ low levels of self-confidence and personal comfort to discuss these issues [36]. The physicians described a large number of discourse barriers including lack of time, workload, and a feeling that this subject was beyond the scope of their expertise and was too intimate [37, 38]. Family physicians expressed frustration at the system’s demand to meet various health measures; therefore, the issue of sexual function was pushed down to the bottom of the list. Family physicians are pressured to deliver an increasing number of preventive services, follow guidelines, engage in evidence-based practice, and deliver patient-centered care; they struggle with how much control they have over their time. Many older adults have more than one chronic disease and one of the greatest challenges for family physicians is the provision of optimal care for older adults with multiple chronic conditions.

When physicians initiated discourse with patients, it mainly revolved around common illnesses. Our study is supported by similar studies in which physicians preferred to discuss sexual dysfunction mainly in combination with old-age risk factors, e.g., hypertension and diabetes, in comparison with patients without risk factors [62]. Coronary heart disease, psychiatric disorders and psychological disorders were found to be disorders in which physicians turn to patients for evaluation of sexual function [63, 64]. It is possible that when the discourse takes place around the focal point of the physician’s expertise, i.e., the physiological field, the family physician feels more confident in making a diagnosis and providing a satisfactory solution. Despite changes in the way older people view sexuality, when they face sexual problems significant barriers to seeking physicians’ help have been identified [65]: some of which relate to the patient (e.g., embarrassment), some to the physician (e.g., ageist attitudes), and others to the geographical or cultural location of the individual (e.g., difficulty in accessing services). Some older people feel more comfortable talking about illness or medication than about their sexual dysfunction [20].

Most sexual complaints brought up in physician-patient discourse - whether through the initiative of the physician or the patient - are related to impotence in men. Consistently, according to the physicians, most patients with sexual dysfunction are men. Studies show that the impotence rate varies according to age, beginning at 2% for men under 40 and going up to 71% for men over the age of 70 [64, 66]. The diagnostic process, which is biological, examines the patient’s symptoms, prevalence of the disorder, background illnesses, medication taken by the patient, and sometimes a physiological evaluation. There are also non-physiological reasons for these difficulties. Psychological problems, such as depression and its related medication, have been associated with sexual dysfunction in older age [20]. However, sexual dysfunctions in older age, due to emotional distress, were less frequently diagnosed and treated compared to young adults [20].

In this study, the treatment administered to male patients was most often identified as PDE5 inhibitor therapy (such as Viagra, Levitra ™ or Cialis ™). This treatment has been described by physicians as first-line, safe and highly effective for sexual dysfunction [67, 68], for use upon demand or on a daily basis [69]. According to an international survey of 12,563 people, only 7% of those who reported ED actually used medication, but 74% claimed they would like to receive medical treatment [70]. In other words, there is a considerable public preference for medical treatment.

Family physicians, in our study, claimed that sexual function has greater significance for men than for women, as the latter have a higher need for intimacy and communication and are more willing to accept a decrease in libido. This is despite studies which indicate a rate of 25–63% of sexual dysfunction among older women, pointing to a lack of estrogen as the main cause [71, 72]. In the English Longitudinal Study of Ageing (ELSA), among 6201 participants (56% women) aged 50 to > 90 [73], compared to men, women of all ages reported lower levels of concern about their sexual activities and functioning, together with lower levels of dissatisfaction with their overall sex lives [74–76]. However, it has been found that physicians treating women with gynecological symptoms recognize a difficulty in sexual functioning, but only very few opt to discuss the matter with them [77]. These findings suggest that family physicians tend not to initiate discourse about sexual functioning with older adults, due to their high workload, time constraints, and fear of offending their patients. Most physicians tended to focus on the physiological aspects of the patient and less on psychological aspects such as intimacy and relationships.

The present study can contribute to the improvement of the family physician’s approach to the subject of sexual functioning in old age. The study emphasizes the numerous barriers which may prevent family physicians from addressing sexual functioning among the population of older adults when it comes to discussing the matter: increased workload, lack of time, fear of offending patients and harming the physician-patient relationship, and the many demands made by the health system, thereby pushing the issue of sexuality down to the bottom of the list of priorities. Although specialization in family medicine is based on the biopsychosocial model, it appears that in the area of sexual functioning and sexual satisfaction in older adults, the social aspect is still somewhat lacking. Based on previous studies, this is a significant issue in the lives of older people, who would like their family physicians to initiate discussions about this subject [3, 78]. The present study indicates that both diagnosis and treatment lean towards the biological end of the biopsychosocial model, and that the focus of communication on the subject is with male patients and on the administration of drug treatment. The research offers practical recommendations for training physicians, including guidance on the importance of sexuality in older age and improving communication with older patients [20, 38], so that family physicians may feel more comfortable about inquiring into the psychosocial aspects, in addition to the biological factors.

It is recommended to ask the patient’s permission to talk about a personal issue [79], for example: “Are you experiencing any difficulty with sexual functioning?”, “What is your sexual orientation?” or “People taking this medication sometimes report problems with sexual function; is that something you are familiar with?” [20]. Another important consideration concerns the use of medication such as Viagra, for instance. Viagra affects more than a man’s erection. It also influences the nature of the sexual relationship that he and his partner share; therefore, we recommended that family physicians include women in the discussion about Viagra and give them an opportunity to be part of the medical consultations and decision-making process regarding a partner’s treatment of erectile difficulties [80].

As the study has demonstrated, a significant barrier is physicians’ heavy workload and busy schedule. Unfortunately, this cannot be easily overcome. Because sexual issues are an important part of general health and are often connected to other medical issues, health policy should advocate educating physicians on how to make time to address and give priority to sexuality. One possible proposal is to create an intervention program to increase physicians’ awareness, so as to be able to identify when they may be sidestepping discussion of a sensitive topic because they are pressed for time or even personally fatigued and overloaded. The program should also legitimize this, and address any guilt, shame or uncomfortable feelings the physician might be feeling or suppressing because he did not give the patient enough time. When the physician is able to recognize this, he can then express to the patient that he realizes he has introduced an important topic. He and the patient can then discuss how they would like to discuss matters further, and encourage him to make an additional appointment in order to give the subject its due attention.In addition, as older people frequently visit their physician accompanied by a family member, physicians should be sensitive in ascertaining whether the patient feels comfortable discussing these issues in their presence [20]. In addition, as older people frequently visit their physician accompanied by a family member, physicians should be sensitive in ascertaining whether the patient feels comfortable discussing these issues in their presence [20].

When most communication concerning sexual difficulties is focused on biological aspects, the message conveyed to patients is that their difficulty is medical, although often this issue, in fact, represents a biopsychosocial problem [40]. Training family physicians in these matters can help them to deal with these barriers and gain a deeper understanding of the topic, thereby increasing their confidence in communicating the issue to their patients. Finally, it is important to acknowledge some of the limitations of the present study. The main limitation of this study is that generalization to broader populations ought to be done with caution. As a qualitative study, we were concerned with generating an in-depth exploration of participants’ understandings and practices, and therefore, the findings presented here are not generalizable. Another limitation is that the research was carried out with a relatively small number of participants - family physicians from Israel. Cultural issues as well as sexual orientation were not directly examined in the present study. An understanding of the impact of cultural and sexual orientation on older adults’ life experiences may assist healthcare professionals in their efforts to determine appropriate interventions. However, this was not the focus of the present study and was not brought up by physicians. There is a need to design curricula to ensure that physicians develop the necessary skills needed to provide comprehensive sexual health care to LGBTQ&I patients. Such curricula include information about sexual orientation and gender identity and tools to address these issues [81].

## Conclusions

Sexual function remains important to older adults and should be recognized as an integral part of their general wellbeing and health. The present study presents the views of 16 family physicians on sexuality in older adults. We found that family physicians have difficulty raising questions about sexuality with older patients, due to workload, time constraints, and fear of offending their patients. Most physicians tended to concentrate on the patient’s medical history, focusing on the physiological aspects, while only a minority of the physicians examined intimacy and marital relations as an integral part of the routine check-up. Family physicians reported that most of the sexuality-related visits were made by men and the main complaint was erectile dysfunction; the treatment most often administered to male patients was PDE5 inhibitor therapy. The current study provides practical recommendations for training family physicians, including stressing the importance of sexuality among the older population, and improving physician-patient communication - conducted with discretion and in complete confidentiality. It is recommended to ask the patient’s permission, at the beginning of the conversation, to discuss a personal issue. It would also be wise to advise patients to schedule a double appointment or an appointment at the end of the day, when the physician is more available.

## Abbreviation

ED: Erectile dysfunction
